# Myth Acceptance Regarding Male-To-Female Intimate Partner Violence amongst Spanish Adolescents and Emerging Adults

**DOI:** 10.3390/ijerph17218145

**Published:** 2020-11-04

**Authors:** Gonzalo Del Moral, Cosette Franco, Manuel Cenizo, Carla Canestrari, Cristian Suárez-Relinque, Morena Muzi, Alessandra Fermani

**Affiliations:** 1 Department of Education and Social Psycology, University Pablo of Olavide, 41013 Seville, Spain; cosette@mentoris.com.es (C.F.); csuarel@upo.es (C.S.-R.); 2 Andalusian Public Health Service, Healt Promotion Area, 41700 Seville, Spain; manuel.cenizo.sspa@juntadeandalucia.es; 3 Department of Education, Cultural Heritage and Tourism, University of Macerata, 62100 Macerata, Italy; carla.canestrari@unimc.it (C.C.); morena.muzi@unimc.it (M.M.); alessandra.fermani@unimc.it (A.F.)

**Keywords:** myth acceptance, male-to-female intimate partner violence, adolescence, dating violence

## Abstract

(1) Background: General beliefs and attitudes toward Male-to-Female Intimate Partner Violence (MFIPV) play a fundamental, critical role in the expression of violent behaviors in relationships during both adolescence and adulthood. The objective of the present study was to contrast the degree of myth acceptance regarding MFIPV, based on the sex and age of Spanish teenagers and emerging adults. (2) Methods: A sample of 1580 participants aged between 15 and 24 took part in the study. The subjects were enrolled in 34 secondary schools and two university centers spread across Seville (Spain). A multivariate analysis of variance (MANOVA) was carried out for the data analysis. (3) Results: Overall, males had a higher level of myth acceptance than females in all the dimensions considered in the study. In the case of significantly high levels of myth acceptance, males quintupled females. The research dimension that revealed the greatest differences was romantic love. Regarding age, a degree of stability was observed in the age period of 15–17 years and 18–20 years, but this subsequently decreased for the age range of 21–24 years. (4) Conclusions: Efforts should be focused on promoting actions to challenge male mandates and narratives concerning romantic or true love.

## 1. Introduction

Dating violence is defined as the broad set of aggressive behaviors (i.e., verbal, psychological, physical and/or sexual) that take place in the relationships of teenage or young couples, whether they be more or less short-lived or long-lasting [1,2]. Although the diversity of this specific concept is quite broad, there are at least two characteristics which single out the particular type of violence which is present in adolescent couples, as opposed to the Male-to-Female Intimate Partner Violence (hereinafter, MFIPV) present in intimate adult relationships. First of all, in specific terms, it happens far more frequently [3] and secondly, it is closely linked to a relationship dynamic characterized by conflict, reciprocity, bidirectionality and power symmetry [4,5]. Therefore, the correlation between violence and victimization is usually high and positive. 

Despite the fact that both males and females seem to act simultaneously as offenders and victims of violence in dating relationships [6,7], it is of upmost importance to direct efforts toward understanding the systemic roots of the male-to-female intimate partner violence (MFIPV) that can be expressed at adolescence and later stages. This is especially important considering the fact that it is a form of violence carried out against women in which the bidirectional character is absent, to such an extent that the asymmetry of power aimed at establishing or perpetuating relations of inequality becomes its defining characteristic [8,9,10].

In fact, the US Centers for Disease Control and Prevention [11] state that intimate partner violence (IPV) starts early and continues throughout the lifespan. When IPV starts in adolescence, it is theoretically called teen dating violence (TDV). About 11 million women who reported MFIPV informed that they first experienced this kind of violence before the age of 18 [12].

In Spain, the national Barometer of the Scopio Project belonging to the National Reina Sofia Centre [13] revealed interesting findings in 2017. For instance, despite the fact that 87% of the young population considers MFIPV as a very serious social problem, 27.4% of the young people surveyed believe that it is normal in couples, while 21.2% think it is an exaggerated politicized issue. The study also adds that the degree of maintenance of these beliefs is higher in the male group than in the female one. The survey on Perception of Gender Violence in Adolescence and Youths from the Spanish Government Delegation for Gender Violence in 2015 [14] showed clearly that one out of every three adolescents and young people, of both sexes, accepts that there may be some kind of control issue in all couples, especially with regard to things one can or cannot say and schedules. In a study of 3043 Spanish teenagers, Donoso, Rubio and Vilá in 2018 [15] added that teenagers and young people perceive the control behaviors which are exercised on the partner in virtual environments as being less violent compared to off-line, real-life situations. In addition, they stated that although the aggressive tendencies are predominantly male, females are responsible for a greater number of aggressions related to the myths of romantic love.

Several theoretical models highlight the importance of certain socio-cultural factors in explaining these key differences between males and females. Stereotyped traits and roles are attributed differently to men and women due to the transmission of sexist attitudinal models and myths about the dynamics of MFIPV, and therefore violence is justified as a way of protecting, educating or dominating the other person in a sentimental relationship [16,17]. In addition, research has shown the close relationship that exists between the assimilation of these types of mythical attitudes and beliefs and actual abuse in relationships [18,19]. Moreover, adolescent males and females with more stereotyped ideas concerning gender roles accept the use of aggression in the dating relationship to a far greater extent and believe that the woman is the one to be assaulted and not the man, in a psychological, physical and sexual manner [20].

In this regard, Bosch and Ferrer (2012) [21] highlight the relevant role played by the acceptance of myths and neomyths [22] regarding MFIPV in the expression, acceptance and justification of violence. According to Peters (2008) [23], these types of myths are defined as rigid beliefs about MFIPV that are widespread, albeit false, and are at the basis of the justification and normalization of violent behaviors and attitudes toward the member of the couple. The main myths are denialist myths (they minimize the severity of MFIPV or even deny it altogether. For example: most reports of battered women are false; men are also victimized; laws criminalize men), myths about marginality (they reduce violence toward certain types of people. For instance: MFIPV violence only happens in multiproblem families or in particular cultures such as Muslim culture, etc.), myths about abusive men (they justify the behavior of men who assault their partners. For example: men who commit MFIPV have problems with alcohol), myths about battered women (they blame women who suffer MFIPV violence. For example: they must have done something to provoke this situation) and myths of romantic love (they describe characteristics that must be present in a true-love relationship, which are related to the acceptance, justification and even desire of certain violent attitudes and behaviors. For example: true love is an excuse for everything, or jealousy is a sign of real love).

All of these myths concerned with MFIPV fulfil individual and social psychological functions beyond the dating relationship and this is what enables the causal attribution process to be carried out—i.e., blaming the victimized woman’s behavior, disqualifying the perpetrator, and minimizing or denying the actual violence [23]. 

The mythical acceptance would also be related to a greater difficulty in carrying out altruistic or heroic actions to help the victimized women [24,25]. The reasoning behind this is based on mythical beliefs that women could avoid abuse if they wanted to, or they must have done something to provoke it in the first place and if they continue in the relationship it is because they are gaining some benefit from it, in any case. 

The work of Bosch and Ferrer (2012) [21] states that if the process of making MFIPV visible and recognizing it as a social problem has been slow and difficult, the maintenance of myths about MFIPV or the emergence of other neomyths can really hinder progress on this issue, which obviously has serious social repercussions. In addition, they also stress the importance of analyzing and contrasting theoretical proposals empirically on this issue and continuing to delve deeper into the relationship between the various mythical beliefs concerning MFIPV. Considering this background, the objective of this study was to contrast the degree of myth acceptance regarding MFIPV in Spanish adolescents and emerging adults, according to sex and age. 

## 2. Materials and Methods 

### 2.1. Participants

The total sample in the study consisted of 1580 adolescents and emerging adults, of which 754 were males and 826 were females, aged between 15 and 24 years (*M* = 17.41; *SD* = 2.05), enrolled in 34 secondary schools in the province of Seville (Spain) and in the Universities of Seville and Pablo de Olavide, coming from the following educational courses and levels: Compulsory Secondary Education (4th year students; *N* = 614), Baccalaureate (1st, 2nd year students; *N* = 498 ), Psychology Degree (1st and 4th year courses; *N* = 252) and Social Work Degree (1st and 4th year courses; *N* = 316). The selection of the participants was carried out through stratified random sampling [26]. The sampling units were provincial district (urban or rural) and school funding (public or private). Statistical analyses showed no significant mean differences in the dependent variables as a result of the specific location of the school and the type of school funding.

Missing data were obtained by the method of imputation by regression. This method assumes that the rows of the data matrix constitute a random sample of a normal multivariate population. For the sample, the average of missing data was 2.6%, and never above 7% for an individual measure. In total, 22 participants obtained scores higher than this percentage and were removed from the database. The multiple imputations with expectation-maximization algorithm technique was used to treat the missing data.

### 2.2. Materials

Myth acceptance relating to MFIPV (hereinafter, MAMFIPV) was captured with the Myth Acceptance Scale based on the Domestic Violence Myth Acceptance Scale [23] and the Spanish map of myths and neomyths about MFIPV [21]. It consisted of 25 items which measure the degree of agreement with various statements which refer to MFIPV, with a response range of 1 to 4 (comprising: strongly disagree, disagree, agree, and strongly agree). An exploratory factorial analysis using the main component method with an Oblimin with Kaiser rotation showed a factorial structure of five dimensions: concept of MFIPV (items 3, 8, 13, 18, 23); gender equality (items 1, 6, 11, 16, 21); romantic love (items 4, 9, 14, 19, 24); characteristics of the male aggressor (items 5, 10, 15, 20, 25); characteristics of the victimized woman (items 2, 7, 12, 17, 22). The “concept of MFIPV” (CONCEPT) dimension evaluates myths related to the definition and characteristics of MFIPV and encompasses some denialist myths and reveals information on the marginality of this kind of violence (for example, “MFIPV occurs more in disadvantaged social classes”). The “gender equality” dimension (EQUAL) evaluates the level of acceptance of the mirage of equality and includes some of the neomyths related to this subject (for example, “nowadays being born a woman has the same advantages as being born a man”). The “romantic love” dimension (ROMLOV) evaluates the degree of agreement with myths related to the concept of true or romantic love (for example, “feeling jealous is proof that you love for your partner”). The fourth dimension called “characteristics of the male aggressor” (MENAGR) assesses the degree of agreement with misconceptions about the stereotype of men who perpetrate MFIPV (e.g., “men who abuse women have problems with alcohol or other drugs”). Finally, the “characteristics of the victimized woman” dimension (WOMVIC) questions myths about women experiencing MFIPV (e.g., “women who are economically independent are not abused by their partners”).

By adding up the scores for each item (having to invert the score of item 3), a score was obtained for each dimension and a total score, with the corresponding correlation in terms of the level of myth acceptance (low, medium, high; see Table 1), was calculated. Higher scores correspond to higher levels of myth acceptance both in dimensions and in the total scale.

In order to establish the validity and reliability of the instrument, McDonald’s Omega (ω), Composite Reliability (*CR*) and Average Variance Extracted (*AVE*) were calculated for each dimension and for the full scale. The psychometric properties of the scale and dimensions are adequate (see Table 2). In exploratory research, values of *CR* between 0.60 and 0.70 are acceptable, while in more advanced stages values have to be higher than 0.70 [27]. The *AVE* value should exceed 0.50 so that it is adequate for convergent validity [28]. Similar with ω, the accepted cut-off value is 0.70, the same as Cronbach’s Alpha [29].

The confirmatory factor analysis showed a good model fit to the data (χ^2^ = 18.35, *df* = 5, *p* < 0.01, CFI = 0.972, and RMSEA = 0.042 (0.022, 0.063)).

### 2.3. Procedure

A letter was sent to the selected secondary schools explaining the objective and purpose of the study and requesting their participation in it. Subsequently, an interview was arranged with the different principals of each school to explain the project in detail and to provide a written informed consent form for parents and students along with a letter detailing the research. In the case of the sample obtained from the two universities, collaboration was requested directly from the students and they were provided with a customized informed consent form. After obtaining the corresponding permits and approval, the questionnaires were handed out.

The set of instruments (this study is part of a larger national research project) was given to the adolescents and young people in their usual classrooms during their regular class period. Each student was given a booklet with all the instruments to be self-managed and at least one member of the research team remained in the classroom during completion to resolve possible doubts.

The study met the requirements of ethical values in human-scale research: informed consent and the right to information, protection of personal data and guarantees of confidentiality, non-discrimination, free of charge and the possibility of abandoning the study at any stage as stated in the Declaration of Helsinki. 

### 2.4. Data Analysis

The data were analyzed using the statistical analysis software IBM SPSS Statistics for Windows, version 25 (IBM Corp., Armonk, NY, USA). First of all, an analysis of the responses to each item by sex was carried out, grouping the response values into two categories: disagreement (comprising “strongly disagree” and “disagree”) and agreement (comprising “strongly agree” and “agree”). The chi-squared test (χ^2^) was used to test hypotheses concerning possible differences in frequency distributions by sex. Secondly, the MAMFIPV levels (low, moderate and high) according to sex were analyzed. Thirdly, the multivariate normality, the equality of variances and the homogeneity of the variance–covariance matrices of the study data were verified. A multivariate factorial analysis of variance (MANOVA) 2 × 3 was carried out to verify the differences in MAMFIPV in the five dimensions (concept of MFIPV, CONCEPT; gender equality, EQUAL; romantic love, ROMLOV; characteristics of the male aggressor, MENAGR; characteristics of the victimized woman, WOMVIC) according to the independent variables of sex (male, female) and age (15–17 years, 18–20 years and 21–24 years) and univariate post hoc tests were applied with the sources of variation in which statistically significant differences were observed with the general multivariate method. Partial eta squared (*η^2^_p_*) was used to estimate the effect size, according to the indications of Cohen [30], in which: large ≥ 0.14, medium ≥ 0.06 and small ≥ 0.01.

## 3. Results

### 3.1. Analysis of Items by Sex

As shown in Table 3, males agreed with myths regarding MFIPV to a greater extent than females in every item except item 12 (“Women who experience MFIPV have a high emotional dependence on their partners”) (χ^2^ = 11.68, *p* < 0.01). There were no significant differences in the frequency of agreement/disagreement between males and females in only three items, namely: item 18 (“MFIPV is unpredictable. Anyone can abuse a partner”) (χ^2^ = 1.54, *n.s.*), item 20 (“Men who use violence against women suffer from psychological problems”) (χ^2^ = 8.21, *n.s.*) and item 22 (“Women who use violence against women have a weak personality”) (χ^2^ = 2.50, *n.s.*).

The three items that obtained the greatest degree of agreement in males are item 19 (“When you have a relationship based on true love, every difficulty can be overcome”), item 2 (“The majority of women who endure an abusive situation could get out of it if they wanted”) and item 1 (“Nowadays, being born a woman has the same advantages as being born a man”). Simultaneously, the three that obtained the greatest degree of disagreement are: item 3 (“IPV only occurs when a man assaults a woman and not the other way around”), item 23 (“MFIPV does not occur in teenage relationships”) and item 7 (“Women who experience MFIPV often engage in certain behaviors that lead to abuse”). 

In the case of females, the three items with the greatest degree of agreement are item 12 (“Women who suffer MFIPV have a high emotional dependence on their partners”), and item 2 and 19 again, similar to the boys. The items that obtained a greater degree of disagreement are the same ones as the boys’ choices (item 3, 7 and 23). Item 3 is an inverted item, so in this case it would indicate that males and females do not agree that MFIPV is only perpetrated by men against women, but that women can also perpetrate MFIPV against men. 

Finally, the three items in which there was a greater difference in the degree of agreement between males and females are item 4 (“Feeling jealous is proof that you actually love your partner”; χ^2^ = 59.28 and *p* < 0.001), item 9 (“For love I would give up and sacrifice other important things related to my personal development”; χ^2^ = 108.80 and *p* < 0.001) and item 14 (“For love I would be willing to change some important things in my personality”; χ^2^ = 120.45 and *p* < 0.001), all of which are related to the myth acceptance of romantic love, that is to say, males would agree more with these statements than females.

### 3.2. MAMFIPV Levels by Sex

Table 4 shows that most males and females have a medium level of MAMFIPV (93.1% and 83.8%, respectively). The number of females with a low level of myth acceptance is three times greater than the number of males and similarly, the number of males with high levels of myth acceptance is almost five times higher than the number of girls (χ^2^ (2, *N* = 1532) = 53.48, *p* < 0.001).

### 3.3. Multivariate Factor Analysis

The MANOVA factor changes the differences in the main effects of sex, Λ = 0.94, *F*(5, 1522) = 19.15, *p* < 0.001, and *η^2^_p_* = 0.06, and age, Λ = 0.94, *F*(10, 3044) = 9.40, *p* < 0.001, and *η^2^_p_* = 0.04. A significant interaction effect was obtained—Λ = 0.99, *F*(10, 3044) = 2.07, *p* < 0.05, and *η^2^_p_* = 0.01.

#### 3.3.1. MAMFIPV Dimensions and Sex

As shown in Table 5, males scored higher on all dimensions of myth acceptance relating to MFIPV, namely: CONCEPT, *F*(1, 1526) = 19.13, *p* < 0.001, *η^2^_p_* = 0.01; EQUAL, *F*(1, 1526) = 29.71, *p* < 0.001, *η^2^_p_* = 0.02; ROMLOV, *F*(1, 1526) = 50.74, *p* < 0.001, *η^2^_p_* = 0.03; MENAGR, *F*(1, 1526) = 10.01, *p* < 0.01, *η^2^_p_* = 0.01, and WOMVIC, *F*(1, 1526) = 23.86, *p* < 0.001, and *η^2^_p_* = 0.02, as well as in the total MAMFIPV, *F*(1, 1526) = 83.53, *p* < 0.001, and *η^2^_p_* = 0.05. 

Moreover, males obtained the highest scores in the myth acceptance of romantic love (*M* = 13.04, *SD* = 2.74) while females did so in myths related to gender equality (*M* = 11.82, *SD* = 2.76).

#### 3.3.2. MAMFIPV Dimensions and Age

As shown in Table 6, no differences were observed in assuming myths about CONCEPT, *F*(2, 1526) = 13.16, *p* < 0.001, *η^2^_p_* = 0.02; EQUAL, *F*(2, 1526) = 3.46, *p* < 0.05, *η^2^_p_* < 0.01; MENAGR, *F*(2, 1526) = 3.64, *p* < 0.05, *η^2^_p_* < 0.01; WOMVIC, *F*(2, 1526) = 13.82, *p* < 0.001, *η^2^_p_* = 0.02, and total MAMFIPV *F*(2, 1526) = 32.25, *p* < 0.001, and *η^2^_p_* = 0.04, between the 15–17 and 18–10 age groups, although both scored significantly higher than the 21–24 age group. Only in relation to ROMLOV was there a significant decrease by age, *F*(2, 1526) = 28.49, *p* < 0.001, and *η^2^_p_* = 0.04, with the highest scores obtained by the 15–17 year-old group, followed by the 18–20 year-old group and, finally, the 21–24 year-old group.

Observing the mean values, the highest level of myth acceptance at 15–17 years was obtained in ROMLOV (*M* = 12.50; *SD* = 2.72), while at 18–20 years (*M* = 12.34; *SD* = 2.71) and 21–14 years (*M* = 11.48; *SD* = 2.51) it was obtained in EQUAL.

#### 3.3.3. Interaction Effect

There was only a significant sex–age interaction effect obtained in WOMVIC dimension—*F*(2, 1526) = 2.57, *p* < 0.05 and *η^2^_p_* < 0.01.

**Figure 1:**
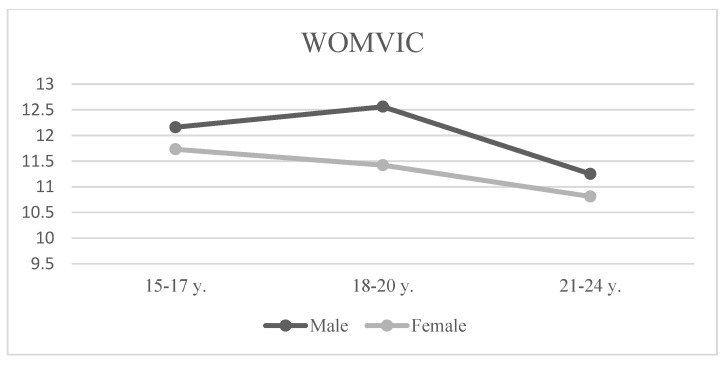
Means in WOMVIC by sex and age.

Older males and females (21–24 years) had the lowest levels of WOMVIC myth acceptance. While females’ scores decreased with age, males’ scores increased and reached the highest values in the 18–20 age group, but were lower in the 21–24-year-old group (see Figure 1).

## 4. Discussion

The objective of this study was to contrast the degree of myth acceptance regarding MFIPV in Spanish adolescents and young people, according to sex and age. Firstly, it should be noted that males have shown a far higher level of myth acceptance than females in all the dimensions contemplated in the study as well as at a general level. Despite most males and females who participated in the study presenting average levels of MAMFIPV, males were five times greater than females in the case of high acceptance levels whereas females were three times greater in the case of a low level of MAMFIPV. These fundamental differences between males and females are observed in all the dimensions of the study and in total myth acceptance. These sex differences converge with recent data from the National Barometer of the Scopio Project belonging to the Spanish Reina Sofia Centre (2017) [13] and with results in other Mediterranean cultural contexts, such as France [31] or Portugal [32].

It is necessary to point out that 90.8% of the participants (males and females) in this study did not agree with the statement “IPV only occurs when a man assaults a woman and not the other way around”, that is to say, they maintain a definition in which IPV can occur from a man to a woman, but also from a woman to a man. In Spain, according to Organic Law 1/2004, of 28 December, gender violence is defined as violence perpetrated against women for the very fact of being so, because they are considered by their aggressors as lacking the minimum rights of freedom, respect and decision-making capacity. The widespread use of gender violence in Spain to refer to IPV has led to it being considered as a one-way problem (male-to-female violence) [33].

However, the Spanish Judiciary General Council (SJGC) (2018a) [34] reported that, of the total precautionary measures registered for the protection of victims of domestic and gender violence between 2011 and 2017, 199.944 corresponded to men who were reported for IPV and 9.067 to women denounced for some form of domestic violence (including under this heading any form of violence exerted in the family environment and not only IPV), a proportion of 22 men for each woman. Regarding homicides, the SJGC (2018b) [35] informed that, between 2003 and 2015, 828 women were killed by their male partner or ex-partner and 72 men by their female partner or ex-partner (92% women and 8% men). Observing these data, it would not be appropriate to affirm the bidirectionality or reciprocity of IPV in terms of equality.

The results of the present study seem to indicate that most young people, males and females, assume a broader definition in which female-to-male IPV may also coexist with MFIPV. These data could be closely related to the characteristics perceived in dating violence by males and females themselves—specifically, conflict, reciprocity, bidirectionality and power symmetry [4,5].

With respect to the differences found according to sex, the only myth in which females score higher than males is the one referring to the emotional dependence of victimized women (“Women who experience MFIPV have a high emotional dependence on their partners”), although both females and males show a high level of myth assumptions about ending the relationship and asking for help (“The majority of women who endure an abusive situation could get out of it if they wanted”). Lelaurain et al. (2019) [31] showed that participants who endorsed more myths also placed more responsibility on the victim of IPV, exonerated the aggressor more, and perceived the violence as less severe. Peters (2008) [23] suggested that MAMFIPV has a defensive function. By blaming the victim, exonerating the aggressor, and discounting the severity of MFIPV, people protect themselves from the psychological threat of perceiving themselves as a potential victim or aggressor. As can be observed in the present study, Spanish females and males endorse myths that blame the victimized woman to a greater extent than the myths that minimize the behavior of the male aggressor.

From these findings it can be stated that in the same and different categories of myths there can exist different levels of assimilation, so that categorically they would not act as a whole. Although Bosch and Ferrer [21,36] claim that myths about MFIPV have a potentiating effect on each other, it might be that a certain level of cognitive dissonance could be maintained even within the same mythical category. Thus, in the category of myths about the victimized woman, it is possible to believe at the same time that women have a high emotional dependence on their partners but that, if they are, they could do something to put an end to the situation (but it is expected that someone very dependent will not possess the agentivity and self-determination to end a relationship).

With respect to age, acceptance levels seem to maintain a certain stability in the period between 15–17 years and 18–20 years, but experience a decrease in the period of 21–24 years, although the group of males in the 18–20 year category shows an increase in scores in the case of myth acceptance relating to the victimized woman. The myth acceptance of romantic love is the only exception to this temporary pattern and decreases significantly depending on age. One aspect that deserves to be highlighted is, precisely, the dimension of myth acceptance with regard to romantic love. The three items in the questionnaire in which there was a greater difference in the degree of agreement between males and females has to do with the myths of romantic love and, in fact, this is the dimension in which males scored highest.

The relationship between the myths of romantic love and the expression and tolerance of dating violence seems to be well founded [37,38,39]. However, romantic love has often been considered as a peripheral aspect of the IPV phenomenon in the literature [31,40]. Gius and Lalli (2014) [41] found that the frame of romantic love is used in the news media to justify the MFIPV and rarely described the males as aggressors. Thus, acceptance of romantic love myths is related to the justification of MFIPV as a male form of loving expression, signs of intimacy or affection rather than a social problem [42,43]. Lelaurain et al. (2019) [31] found that behind the adherence to romantic love, the legitimization of IPV is determined by the internalization of patriarchal ideologies defining gender-symbolic roles and justifying male domination. From this point of view, a greater acceptance of myths of romantic love by males would be related to a greater tolerance and justification of the MFIPV.

The findings of this study may allow us to reflect on the results obtained recently by Donoso et al. (2018) [15] who state that females show more aggressive behavior in violence related to the myths of romantic love than males do. Although several studies have shown that males have a higher level of acceptance of romantic love myths [37,44], they may not act in the same way in the expression of violence in dating relationships based on gender. In other words, males currently assume, to a greater degree, the myths of romantic love but females attack more based on them, using traditionally masculine forms [45].

It is a well-known fact that there is much greater social tolerance of low-intensity physical aggression (such as slapping or pushing) when these minor acts are perpetrated by girls or women [46,47]. In cases where female aggression is related to one of the myths of romantic love even adopting a traditionally masculine form, it may find a new form of social justification similar to that carried out amongst men who perpetrate MFIPV. In fact, Oberst, Chamorro and Renau (2016) [48] state that identification with male traits is associated with a greater degree of psychological well-being in both adolescent males and females.

## 5. Conclusions

The implications of these findings are of great interest for the prevention of MFIPV. First of all, myths are organized in such a way that deactivating a belief in a mythical dimension does not imply questioning the rest of the myths in that category, so it would be interesting to work at the level of cognitive maps or at least at a categorical level rather than with individual myths. Secondly, it is believed to be necessary to develop educational programs specially designed for males since the level of MAMFIPV is significantly higher amongst them than it is amongst females. In particular, efforts should be focused on promoting actions to challenge male mandates and narratives concerning romantic or true love. Thirdly, one of the most attractive proposals is to work through strengthening the pro-social behavior of helping, as opposed to being bystanders of MFIPV. This work proposes intervention with heroic imagination [49] in such a way that males and females can put themselves in a different situation with regard to the problem, in which they feel that MFIPV is something they can intervene in, diminishing the feeling that it is something that only belongs to the adult world. Fourthly, it is important to note that certain indicators seem to improve with age, with a clear decrease in mythical acceptance at 21–24 years of age.

This last point, however, is related to one of the main limitations of the study: the transversal nature of the design prevents the establishment of a causal relationship between the variables studied, so longitudinal studies would be necessary to deepen the findings obtained. In fact, the improvement in the 21–24 year-old group could be due to the fact that a considerable number of adolescents drop out of secondary education at the age of 16, while those with the greatest number of protective factors remain in education. Even so, the results of this study enable us to advance and make firm progress in the understanding of certain socio-cultural elements that seem to be playing an important role in the attitudes and behaviors toward MFIPV that are strengthened in adolescence and that could intervene decisively in later stages of development.

## Figures and Tables

**Table 1:** Levels of myth acceptance por the dimensions and the scale.

Dimension Score	Full Scale Score	MAMFIPV Level
5–10	25–50	Low
11–14	51–74	Medium
15–20	75–100	High

MAMFIPV = Myth Acceptance relating to MFIPV.

**Table 2:** Reliability, validity indexes, mean, and standard deviation.

Dimensions	ω	*CR*	*AVE*	*M*	*SD*
MAMFIPV Total Scale	0.976	0.96	0.52	57.49	7.11
CONCEPT	0.911	0.83	0.52	9.59	4.24
EQUAL	0.911	0.83	0.50	12.21	2.72
ROMLOV	0.914	0.87	0.58	12.17	7.61
MENAGR	0.910	0.83	0.50	11.6	2.41
WOMVIC	0.900	0.84	0.52	11.84	4.13

ω = McDonald’s Omega; *CR* = Composite Reliability; *AVE* = Average Variance Extracted; *M* = Mean; *SD* = Standard Deviation.

**Table 3:** Scores by item grouping disagreements and agreements according to sex. Percentages and chi-square.

Items	Disagreement		Agreement		χ^2^	*p*
	Male	Female	Male	Female		
1. Nowadays, being born a woman has the same advantages as being born a man.	*N* = 260 % = 16.5	*N* = 341 % = 21.6	*N* = 493 % = 31.2	*N* = 485 % = 30.7	9.68	0.02 *
2. The majority of women who endure an abusive situation could get out of it if they wanted.	*N* = 222 % = 14.1	*N* = 314 % = 19.9	*N* = 531 % = 33.6	*N* = 511 % = 32.4	18.29	0.000 ***
3. IPV only occurs when a man assaults a woman and not the other way around.	*N* = 667 % = 42.3	*N* = 765 % = 48.5	*N* = 85 % = 5.4	*N* = 59 % = 3.8	19.13	0.000 ***
4. Feeling jealous is proof that you actually love your partner.	*N* = 382 % = 24.2	*N* = 564 % = 35.7	*N* = 371 % = 23.5	*N* = 261 % = 16.6	59.28	0.000 ***
5. Men who mistreat women have problems with alcohol or other drugs.	*N* = 503 % = 32.0	*N* = 664 % = 42.2	*N* = 247 % = 15.7	*N* = 160 % = 10.1	41.36	0.000 ***
6. Currently, men and women have the same opportunities to advance in their working life.	*N* = 311 % = 19.7	*N* = 427 % = 27.0	*N* = 442 % = 28.0	*N* = 399 % = 25.3	28.79	0.000 ***
7. Women who experience MFIPV often engage in certain behaviors that lead to abuse.	*N* = 618 % = 39.2	*N* = 770 % = 48.8	*N* = 134 % = 8.5	*N* = 55 % = 3.5	139.41	0.000 ***
8. MFIPV are isolated acts that occur as a result of the relationship.	*N* = 529 % = 33.6	*N* = 689 % = 43.7	*N* = 223 % = 14.2	*N* = 134 % = 8.5	59.00	0.000 ***
9. For love I would give up and sacrifice other important things related to my personal development.	*N* = 468 % = 29.6	*N* = 694 % = 44.0	*N* = 284 % = 18.1	*N* = 131 % = 8.3	108.80	0.000 ***
10. Men who mistreat women are sons of fathers who mistreated their wives.	*N* = 557 % = 35.5	*N* = 660 % = 42.0	*N* = 196 % = 12.4	*N* = 158 % = 10.1	27.12	0.000 ***
11. Currently, women and men spend the same time caring for the children.	*N* = 498 % = 31.4	*N* = 634 % = 40.2	*N* = 256 % = 16.2	*N* = 192 % = 12.2	30.31	0.000 ***
12. Women who experience MFIPV have a high emotional dependence on their partners.	*N* = 275 % = 17.4	*N* = 245 % = 15.5	*N* = 477 % = 30.2	*N* = 651 % = 36.9	11.68	0.009 **
13. MFIPV occurs more in disadvantaged social classes.	*N* = 509 % = 32.4	*N* = 639 % = 40.5	*N* = 242 % = 15.3	*N* = 186 % = 11.8	28.31	0.000 ***
14. For love I would be willing to change some important things in my personality.	*N* = 375 % = 23.7	*N* = 622 % = 38.7	*N* = 379 % = 24.0	*N* = 214 % = 13.6	120.45	0.000 ***
15. Men who mistreat women do so because they have trouble controlling their impulses.	*N* = 351 % = 22.2	*N* = 455 % = 28.8	*N* = 403 % = 25.5	*N* = 370 % = 23.5	18.71	0.000 ***
16. Currently, women and men have the same opportunities to access the labor market.	*N* = 334 % = 21.2	*N* = 471 % = 29.8	*N* = 420 % = 26.5	*N* = 355 % = 22.5	27.71	0.000 ***
17. Women who have economic independence do not suffer abuse from their partner.	*N* = 580 % = 36.8	*N* = 713 % = 45.2	*N* = 170 % = 10.8	*N* = 113 % = 7.2	29.62	0.000 ***
18. MFIPV is unpredictable. Anyone can abuse a partner.	*N* = 392 % = 24.9	*N* = 416 % = 26.4	*N* = 361 % = 22.9	*N* = 405 % = 25.8	1.54	0.67 *n.s.*
19. When you have a relationship based on true love, every difficulty can be overcome.	*N* = 162 % = 10.3	*N* = 234 % = 14.8	*N* = 592 % = 37.5	*N* = 591 % = 37.4	23.43	0.000 ***
20. Men who use violence against women suffer from psychological problems.	*N* = 293 % = 18.7	*N* = 340 % = 21.6	*N* = 460 % = 29.1	*N* = 482 % = 30.6	8.21	0.08 *n.s.*
21. Currently, men and women share housework equally.	*N* = 458 % = 29.0	*N* = 598 % = 37.8	*N* = 296 % = 18.7	*N* = 227 % = 14.5	32.91	0.000 ***
22. Women who suffer violence have a weak personality.	*N* = 326 % = 20.7	*N* = 344 % = 21.7	*N* = 426 % = 27.0	*N* = 482 % = 30.6	2.50	0.47 *n.s.*
23. MFIPV does not occur in teenage relationships.	*N* = 645 % = 40.9	*N* = 784 % = 49.8	*N* = 108 % = 6.8	*N* = 39 % = 2.5	67.77	0.000 ***
24. Sharing everything with the partner is a sign of true love.	*N* = 264 % = 16.8	*N* = 412 % = 26.1	*N* = 489 % = 31.0	*N* = 414 % = 26.1	47.76	0.000 ***
25. Men who mistreat women are socially violent.	*N* = 370 % = 23.5	*N* = 450 % = 28.5	*N* = 383 % = 24.3	*N* = 376 % = 23.7	8.70	0.03 *

*** *p* < 0.001; ** *p* < 0.01; * *p* < 0.05; *n.s.*: no significant.

**Table 4:** MAMFIPV levels according to sex. Percentages and chi-square.

Sex	MAMFIPV				χ^2^	*p*
		Low	Medium	High		
Male	*N*	37	684	14	53.48	0.000 ***
	%	5.0	93.1	1.9		
Female	*N*	126	668	3		
	%	15.8	83.8	0.2		
Total	*N*	163	1352	17		
	%	10.6	88.3	1.1		

*** *p* < 0.001.

**Table 5:** Means, standard deviations, F values and effect size between sex and the variables under study.

Dimensions	Sex		*F*(1, 1526)	*η^2^_p_*
	Male	Female		
CONCEPT	11.91 (1.91) ^a^	11.23 (1.71) ^b^	19.13 ***	0.01
EQUAL	12.68 (2.60) ^a^	11.82 (2.76) ^b^	29.71 ***	0.02
ROMLOV	13.04 (2.74) ^a^	11.44 (2.56) ^b^	50.74 ***	0.03
MENAGR	11.87 (2.44) ^a^	11.36 (2.36) ^b^	10.01 **	0.01
WOMVIC	12.20 (2.08) ^a^	11.53 (1.93) ^b^	23.86 ***	0.02
Total MAMFIPV	61.70 (6.47) ^a^	57.37 (6.55) ^b^	83.53 ***	0.05

*** *p* < 0.001; ** *p* < 0.01; ^a^, ^b^: post-hoc comparisons values, a > b, Note: max/min scores by dimensions (5–20) and total scale (25–100). Concept of MFIPV (CONCEPT) (items 3, 8, 13, 18, 23); gender equality (EQUAL) (items 1, 6, 11, 16, 21); romantic love (ROMLOV) (items 4, 9, 14, 19, 24); characteristics of the male aggressor (MENAGR) (items 5, 10, 15, 20, 25); characteristics of the victimized woman (WOMVIC) (items 2, 7, 12, 17, 22).

**Table 6:** Means, standard deviations, F values and effect size between age and the variables under study.

Dimensions	15–17 y.	Age		*F*(2, 1526)	*η^2^_p_*
		18–20 y.	21–24 y.		
CONCEPT	11.69 (1.84) ^a^	11.49 (1.92) ^a^	10.84 (1.41) ^b^	13.16 ***	0.02
EQUAL	12.31 (2.73) ^a^	12.34 (2.71) ^a^	11.48 (2.51) ^b^	3.46 *	0.00
ROMLOV	12.50 (2.72) ^a^	12.12 (2.87) ^b^	10.60 (2.19) ^c^	28.49 ***	0.04
MENAGR	11.64 (2.43) ^a^	11.75 (2.46) ^a^	11.03 (2.07) ^b^	3.64 *	0.00
WOMVIC	11.94 (1.97) ^a^	12.00 (2.14) ^a^	10.95 (1.91) ^b^	13.82 ***	0.02
Total MAMFIPV	60.08 (6.68) ^a^	59.70 (7.09) ^a^	54.90 (5.58) ^b^	32.25 ***	0.04

*** *p* < 0.001; * *p* < 0.05; ^a^, ^b^, ^c^: post-hoc comparisons values, a > b > c.
